# Cold Feet

**Published:** 1883-03

**Authors:** 


### COLD FEET.

[*Translated by the Editor from the Journal d'Hygiene.*]

We often hear persons complain of having cold feet, not only in the winter but at all seasons of the year. We do not understand by that feet that become cold accidentally by standing in the street, on the cold ground, on the snow or on the ice, but we wish to speak of a condition of disease which consists in having the feet habitually cold. If a person cannot have the feet warm, in spite of warm felt shoes, in spite of woolen stockings, and these moreover in a warm room, and his feet are cold in bed during the night, he is in a condition of chronic malady, which is the cause of many other maladies. What is the cause of such cold feet ? The physiologist would say that animal heat depends on the blood which gives out its heat to all parts of the body, and if the circulation is sluggish in any part, there is in that part a sensation of cold. In that chronic condition which consists in having the feet cold we have then a defective distribution of the blood. As the cold feet do not receive a sufficient quantity of blood, therefore they do not get a sufficient quantity of warmth, the nutrition of these extremities is perverted, some of their functions are arrested and organic troubles follow. Not only the part affected becomes diseased, but as a result other remote organs.

To have the feet habitually cold is not the result of a deficient quantity of blood in the system ; the quantity exists, but it is blocked up in other places, in the arteries and veins.

This ebbing tide of blood, which sometimes causes extravasations of blood has often been followed by dangerous symptoms. Thus we find hemorrhoids in men, and nearly all uterine affections in women, are due to habitually cold feet, and when these cease, the affections that follow disappear also.

Habitually cold feet are the origin of many affections of the stomach. This unequal circulation of the mass of the blood often causes diseases of the liver, of the intestines, of inflammations and catarrhs of the stomach. This blood repelled from the extremities goes very often to congest the lungs, organs which easily yield to sanguine congestions. And it can be said that ninety times in one hundred, diseases of the lungs are due to cold feet. Cold feet often induce difficult respiration and asthma ; the heart becomes subject to palpitations. The congestion reaches the larynx, the head ; from these the trouble extends to the brain and to the eyes.

All these affections disappear when the feet are kept warm.

It is to our own carelessness that habitually cold feet are due. We systematically render ourselves ill. Even from the cradle they raise us to have cold feet. The stockings are thin and the shoes narrow with elastic tips, so that the blood cannot circulate in the feet ; add to this the evil custom of having garters, and the general want of care for the feet, and it is easily seen why these organs revenge this treatment later.

What is necessary to avoid cold feet, and to cure this infirmity when it exists?

It is not well to give baths too warm to children ; it is well to continue baths at all ages. The feet should be attended to, and infants should have loose shoes, without elastics, and then the feet will keep warm. If the feet have become habitually cold, it is neces sary to have patience and not think that a trouble that has required twenty or thirty years to establish can be cured in one night.

There is no specific for the cure of chronic cold feet. The cure of the evil pertains to natural therapeutics, as rubbing, vapor baths and walking. In this way warmth comes to the feet, and with it health returns. When the feet are warm it becomes easy to talk, for then the head is cool and the blood circulates freely. The old proverb which said that "head cool, feet warm and waist free may laugh at the doctors" finds here confirmation.

An excellent vapor bath for the feet is made thus :

In a small box put a jug of boiling water, and envelop this jug with cloths wet with hot water. Place the naked feet on slats that cover the box, and then envelop them with flannel. The vapor which rises from the wet cloths warms the feet and dilates the blood vessels; thus the blood has more space to circulate, and the nerves are excited to action. After a certain time wipe the feet with a dry towel. It is well also to have at the feet, in the bed, during the night, a bottle or jug filled with hot water.

---

WHILE putting on a clean shirt a Hartwell, Ga., man fell over a trunk and broke his collar-bone. But he says he's going to try it again when he gets well.—*Boston Post.*

